# Brazilin from *Caesalpinia sappan* L. as a Proprotein Convertase Subtilisin/Kexin Type 9 (PCSK9) Inhibitor: Pharmacophore-Based Virtual Screening, *In Silico* Molecular Docking, and *In Vitro* Studies

**DOI:** 10.1155/2023/5932315

**Published:** 2023-10-11

**Authors:** Muhammad Iqbal, Nur Hasanah, Aimee Detria Arianto, Widya Dwi Aryati, Meidi Utami Puteri, Fadlina Chany Saputri

**Affiliations:** ^1^Postgraduate Program, Faculty of Pharmacy, Universitas Indonesia, UI Depok Campus, Jakarta, West Java 16424, Indonesia; ^2^Pharmacy Department, Widya Dharma Husada School of Health Science, South Tangerang, Banten 15417, Indonesia; ^3^Laboratory of Biomedical Computation and Drug Design, Faculty of Pharmacy, Universitas Indonesia, UI Depok Campus, Jakarta, West Java 16424, Indonesia; ^4^Department of Pharmacology-Toxicology, Faculty of Pharmacy, Universitas Indonesia, UI Depok Campus, Jakarta, West Java 16424, Indonesia; ^5^National Metabolomics Collaborative Research Center, Faculty of Pharmacy, Universitas Indonesia, UI Depok Campus, Jakarta, West Java 16424, Indonesia

## Abstract

**Background:**

Proprotein convertase subtilisin/kexin type 9 (PCSK9) is a crucial regulator of low-density lipoprotein cholesterol (LDL-c) levels, as it binds to and degrades the LDL receptor (LDLR) in the lysosome of hepatocytes. Elevated levels of PCSK9 have been linked to an increased LDL-c plasma levels, thereby increasing the risk of cardiovascular disease (CVD), making it an attractive target for therapeutic interventions. As a way to inhibit PCSK9 action, we searched for naturally derived small molecules which can block the binding of PCSK9 to the LDLR.

**Methods:**

In this study, we carried out *in silico* studies which consist of virtual screening using an optimized pharmacophore model and molecular docking studies using Pyrx 0.98. Effects of the candidate compounds were evaluated using *in vitro* PCSK9-LDLR binding assays kit.

**Results:**

Eleven natural compounds that bind to PCSK9 were virtually screened form HerbalDB database, including brazilin. Next, molecular docking studies using Pyrx 0.98 showed that brazilin had the highest binding affinity with PCSK9 at −9.0 (Kcal/mol), which was higher than that of the other ten compounds. Subsequent *in vitro* PCSK9-LDLR binding assays established that brazilin decreased the binding of PCSK9 to the EGF-A fragment of the LDLR in a dose-dependent manner, with an IC_50_ value of 2.19 *μ*M.

**Conclusion:**

We have identified brazilin, which is derived from the *Caesalpinia sappan* herb, which can act as a small molecule inhibitor of PCSK9. Our findings suggest that screening for small molecules that can block the interaction between PCSK9 and the LDLR *in silico* and *in vitro* may be a promising approach for developing novel lipid-lowering therapy.

## 1. Introduction

Cardiovascular disease (CVD) continues to be the primary cause of morbidity and mortality worldwide, with atherosclerosis being identified as the main risk factor [1]. Despite advances in CVD prevention and treatment, atherosclerosis remains a significant public health concern and requires continued efforts to mitigate its impact [1]. Biomarkers such as total cholesterol, low-density lipoprotein cholesterol (LDL-c), high-density lipoprotein cholesterol (HDL-c), and triglycerides (TG) are well-established indicators of atherosclerotic cardiovascular disease (ASCVD) [2, 3]. While the precise role of TG in ASCVD is still debated in terms of its causal relationship, the significance of LDL-c in the initiation and progression of ASCVD is widely acknowledged [2, 3]. Furthermore, multiple studies have consistently indicated a strong association between elevated LDL-c levels and the development of atherosclerotic cardiovascular disease (ASCVD) [1, 3]. Therefore, reducing LDL-c levels has emerged as a primary target for preventing the onset and progression of ASCVD [1, 3]. Statin drugs are currently the primary agents used to lower levels of LDL-c [4]. The cholesterol-lowering effect of statins has been consistently associated with a lower risk of cardiovascular events [4, 5]. However, in many cases, residual risk remains due to the inability to achieve desirable LDL-c levels or the presence of other traits that predispose individuals to CVD [6–8]. Therefore, alternative or adjunctive lipid-lowering therapies are being extensively investigated by searching for novel molecules or pathways that can be targeted, such as of proprotein convertase subtilisin/kexin type 9 (PCSK9) [9].

Extensive research has been conducted on the function of PCSK9 due to its involvement in cholesterol metabolism and cardiovascular diseases [10–12]. It is reported that PCSK9 targets LDL receptors (LDLR) for degradation in lysosomes [11]. Binding occurs between PCSK9 and LDLR on the surface of hepatocyte cells, leading to the formation of a PCSK9/LDLR complex that enters the cell through clathrin-coated [11]. Prior studies have established that the interaction between PCSK9 and LDLR takes place via the catalytic domain of PCSK9 and the epidermal growth factor precursor homology repeat-A (EGF-A) domain of LDLR [13]. By increasing LDL-c levels in plasma, PCSK9 plays a crucial role in controlling plasma cholesterol levels [11, 12]. PCSK9 deficiency has been shown to improve the survival rate of CVD patients, while gain- and loss-of-function mutations in PCSK9 have significant effects on hyper- or hypocholesterolemia in individuals and impact the development of ASCVD [14]. Additionally, studies on PCSK9 knockout mice have revealed that removing PCSK9 leads to hypocholesterolemia, reduced atherosclerosis development, and increased sensitivity to statin treatment [15, 16].

In an effort to inhibit the PCSK9's expression as well as its interaction with LDLR, monoclonal antibodies and small peptide inhibitors targeting PCSK9 have been developed [11]. The US FDA has approved two monoclonal antibodies, alirocumab and evolocumab, that have shown success in decreasing LDL-c levels in patients with familial hypercholesterolemia, intolerance to statins, or a major risk of cardiovascular disease who are unable to control their LDL-c levels with statins or ezetimibe [17, 18]. Despite this, currently available PCSK9 inhibitors have limitations due to their high cost and inconvenient method of administration [18]. As a result, the search for small molecule PCSK9 inhibitors continues.

Pfizer has claimed to have found a PCSK9 antisecretagogue in the form of a small molecule, but the research has not moved forward to clinical trials [19, 20]. Other companies such as Genentech and Portola Pharmaceuticals have also identified potential PCSK9 inhibitors, including small peptides and patented compounds [21, 22]. However, none of these have advanced past the discovery stage, and there is a limited amount of information available about them. To address the need for an effective and affordable lipid-lowering therapy, researchers are exploring the potential of natural products as PCSK9 inhibitors [23]. Therefore, this study aims to identify natural products, particularly from Indonesia that can act as specific PCSK9 inhibitors as alternative or adjunctive lipid-lowering therapy.

Exploiting computational techniques such as ligand-based virtual screening is a rapid approach to discover compounds with the intended pharmacological properties [24–26]. Pharmacophore modeling, which utilizes a 3D structure that encodes the meaningful interaction between ligand and target, can effectively filter potential compounds in large quantities with accuracy and speed [24–26]. With access to the HerbalDB database containing information on compounds from numerous herbal plants in Indonesia, eleven natural compounds were selected as potential candidates for PCSK9 inhibitor, including morindone, gentisin, mesuaxanthone A, beta-phenethylamine, brazilin, pterofuran, n-cis-feruloyltyramine, bethanidine, 6-methoxykaempferol, gartanin, and alizarin, which meet Lipinski's rule-of-five. Using molecular docking studies with Pyrx 0.98, brazilin exhibited the highest binding affinity at −9.0 (Kcal/mol) compared to the other ten compounds. In consistent with the *in silico* studies, *in vitro* PCSK9-LDLR binding assay showed that brazilin decreased the interaction of PCSK9 to the EGF-A fragment of the LDLR, with an associated IC_50_ of value 2.19 *μ*M.

Collectively, this study highlights the discovery of a small molecule inhibitor, brazilin, derived from *Caesalpinia sappan* herb that hamper the PCSK9-LDLR interaction based on both *in silico* and *in vitro* studies.

## 2. Materials and Methods

### 2.1. Construction of Ligand Conformations and Pharmacophore Models

The structures of the selected compounds were constructed using MarvinSketch software and saved in ^*∗*^.mol format. Pharmacophore models were constructed using LigandScout software.

### 2.2. Pharmacophore Model Optimization and Validation

LigandScout was used to optimize and validate pharmacophore models. The active and decoy set databases are test sets for screening pharmacophore models. The selection of the test compounds (test set) was carried out by grouping the compounds using ChemMine Tools. The decoy compound was downloaded from DUD-E (https://dude.docking.org). The parameters measured are the receiver operating characteristic (ROC) curve which consists of the area under the curve (AUC), sensitivity and specificity, as well as the enrichment factor (EF) value.

### 2.3. Virtual Screening Based on a Pharmacophore Model

Virtual screening was performed using LigandScout. Active compounds were downloaded from HerbalDB (https://herbaldb.farmasi.ui.ac.id/) [26]. The optimized pharmacophore model was selected for virtual screening. The screening results were candidate compounds (hits) sorted based on the best pharmacophore-fit score.

### 2.4. Evaluation of Physicochemical Properties of Virtual Screening Results

Physicochemical properties of compound active from *Caesalpinia sappan* using KNIME analytics platform with the extension “Knime Community Extensions-Chemminformatics” were evaluated using the Lipinski's rule-of-five node.

### 2.5. Molecular Docking

#### 2.5.1. 3D Structure of the Target Protein (PCSK9)

Protein data for PCSK9 3D structure were obtained from the Protein Data Bank (https://www.rcsb.org), which is an online database of protein structures. The interaction between PCSK9 and LDLR occurs at the C-terminal of PCSK9 and the EGF-A domain of LDLR [27]. The prodomain and C-terminal domain of PCSK9 play a critical role in this interaction, which leads to increased plasma LDL-C levels and a higher risk of cardiovascular disease [28]. Out of all the available protein structures in the PDB database, PDB ID 6U26 [29] was selected as the final structure because of its high resolution (1.59 Å) and active site domains. The protein structure was separated from water molecules and native ligands using the BIOVIA application (https://discover.3ds.com/discovery-studio). Furthermore, protonation is carried out on macromolecules (receptors) using Discovery Studio software by adding hydrogen polar atoms. The active compound was compared to the native ligand attached to macromolecules, which allowed for a better understanding of the interactions between the compound and PCSK9.

#### 2.5.2. Ligand Preparation

3D structures of active compound were downloaded from the protein database (https://www.rcsb.org) and have been converted to pdb format. Furthermore, in the ligands, energy minimization is carried out and the pdb ligand structure is converted to pdbqt. The Swiss PDB viewer used the Gromos 43BI force field to minimize macromolecular structures, while ligand energy minimization used UCSF Chimera with the AMDERfFsb force field.

#### 2.5.3. Preparation of Ligand Cocrystal as Comparison

The preparation of ligand cocrystals for comparison involved extracting cocrystal ligands from the 3D structure of macromolecules (PDB ID: 6U26). This separation from the protein was achieved using the BIOVIA application available at https://discover.3ds.com/discovery-studio. These cocrystal ligands served as a reference for comparing with the candidate compounds. For the visualization of interactions between compounds and amino acids, the BIOVIA application available at https://discover.3ds.com/discovery-studio was employed.

#### 2.5.4. Identification of the PCSK9 Active Site

We selected the active site of PCSK9 (PDB ID 6U26) where it interacts with LDLR. It was located between pocket allosteric at the amino acids Asp360, Cys358, Arg 357, Arg476, Arg 458, and Pro331 [30, 31].

#### 2.5.5. Simulation of Molecular Docking and Visualization of Docking Results

Molecular docking simulations were conducted using AutoDock Vina through PyRx 0.98 software to evaluate the interaction affinity between candidate compounds and the target protein PCSK9. The coordinates utilized for docking were set as follows: center (x: 39.643, y: 29.204, z: 29.773), and dimensions (Å): x: 30.230, y: 30.230, z: 30.230. For the visualization of interactions between compounds and amino acids, the BIOVIA application available at https://discover.3ds.com/discovery-studio was employed.

### 2.6. PCSK9-LDLR Binding Assay

The brazilin used in the experiment was obtained from Solarbio (Solarbio Life Sciences, Beijing, China). The *Caesalpinia sappan* extract used in the experiment was obtained from Phytochemindo (PT. Phytochemindo Reksa, Bogor, Indonesia). Concentrations of brazilin were prepared at 12.5, 6.25, 3.13, 1.56, 0.78, and 0.39 *μ*g/mL and were dissolved in a conjugate dilution buffer to reach the desired concentrations. Similarly, *Caesalpinia sappan* extract concentrations of 12.5, 6.25, 3.13, 1.56, 0.78, and 0.39 *μ*g/mL were prepared using the same method. Samples were screened for their inhibitory activity against the PCSK9-LDLR interaction using a CircuLex PCSK9-LDLR *in vitro* binding assay kit (MBL International, Woburn, MA, USA) according to the manufacturer's instructions. In brief, a 10x concentration working solution was prepared by dissolving 100 mL in 900 mL of high-purity water. The His-tagged recombinant PCSK9 wild type was reconstituted to a stock solution of 2 *μ*g/mL by double-distilled water. To start the assay, 100 *μ*L of the His-tagged PCSK9 solution and the test compound were added to each well of a 96-well microplate. The microplate was then incubated at room temperature for 2 hours, with shaking at 300 rpm on an orbital microplate shaker. Following this, 100 *μ*L of biotinylated monoclonal antibody was added to each well, and the microplate was again incubated at room temperature for 2 hours with shaking. Next, 100 *μ*L of HRP-conjugated streptavidin was added to each well, and the microplate was incubated at room temperature for 2 hours with shaking. Each step was followed by washing the microplate four times with 350 *μ*L of wash buffer. After the final wash step, 100 *μ*L of substrate reagent was added to each well, and the microplate was incubated at room temperature (25°C) for 20 minutes with shaking. The reaction was then stopped by adding 100 *μ*L of stop solution to each well. The absorbance of each well was measured at 450 nm using a plate reader. The absorbance data obtained from the assay was analyzed using GraphPad Prism 9 (GraphPad Prism, USA) software. The data were converted into the form of percent inhibition of PCSK9-LDLR binding, and the IC_50_ value of each test compound was obtained from six different concentrations of the test group.

## 3. Results and Discussion

### 3.1. Virtual Screening Based on a Pharmacophore Model

**Figure 1:**
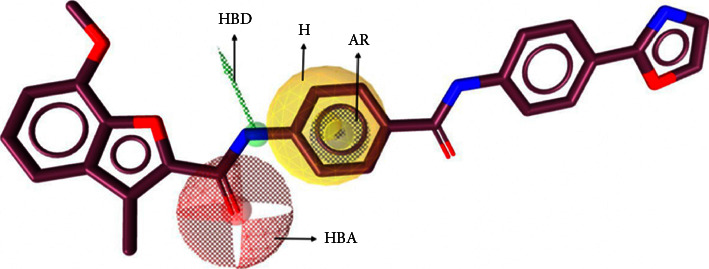
Best pharmacophore features.

When the structure of the target protein is well established, structure-based screening of small molecules that target protein-protein interaction is a useful tool in drug development [32]. In this regards, computational approaches have become an essential tool and virtual screening using pharmacophore modeling is one such approach. This method involves using the 3D structure of the molecule to screen a large number of potential compounds accurately and rapidly by encoding the critical interactions between the ligand and the target [32]. A total of 10 pharmacophore models were obtained. Determination of the best pharmacophore model was based on the score obtained. The higher the value, the better fit the model [32]. In our study, we have optimized and validated our pharmacophore model to filter out potential compounds (Figure 1 and Table 1). Our results showed that the model we selected from the screening process correctly identified active compounds (TP) and inactive compounds. Based on our virtual screening results, we have identified potential compounds from HerbalDB with the highest hit rate. These compounds include brazilin, morindone, alizarin, gartanin, mesuaxanthone A, 6-Methoxykaempferol, gentisin, beta-phenethylamine, n-Cis-feruloyl tyramine, pterofuran, and bethanidin (Table 2).

Furthermore, the active compound we identified has a molecular weight (MW) of less than 500 Dalton, a logP value of less than 5, and a hydrogen bond donor (HBD) value of less than 5. Additionally, the hydrogen bond acceptor (HBA) value is less than 10 (Table 3). The MW of a drug is related to its distribution process inside the body [33]. Drugs with a MW of more than 500 Dalton are relatively large and can make it difficult for them to penetrate biological membranes, leading to poor absorption and distribution [33]. Our active compound has a smaller MW, making it easier to penetrate biological membranes and absorb more efficiently, resulting in faster absorption and distribution. The logP value, which measures the lipophilicity of the compound, and HBD value indicate the ability of the compound to be absorbed by the intestine and have good biological activity [33]. Taken together, the physicochemical properties of the active compound identified from our virtual screening suggest that it has the potential to be easily absorbed, distributed and has good biological activity, making it a promising candidate for further drug development.

### 3.2. Molecular Docking

**Figure 2:**
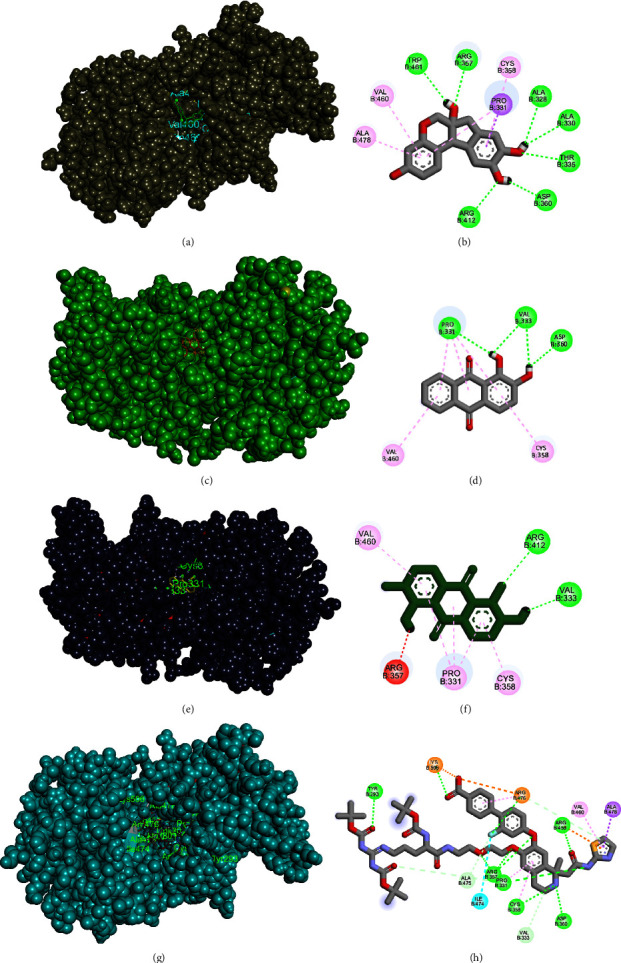
The visualization of the best molecular docking results from candidate compounds. (a, b) Brazilin-PCSK9 (−9.0 kcal/mol). (c, d) Alizarin-PCSK9 (−8.4 kcal/mol). (e, f) Morinda-PCSK9 (−8.4 kcal/mol). (g, h) Ligand cocrystal (−8.4 kcal/mol). Interactions are represented in color, purple: pi-sigma; green: conventional hydrogen bond; light green: carbon hydrogen bond; turqoise: halogen (fluorine); red: unfavorable donor-donor; pink: alkyl and pi-alkyl; and orange: pi-cation.

We further investigated the binding affinity of the active compound, brazilin, with PCSK9 using molecular docking simulations using Vina autodock in Pyrx 0.98. Our results (Table 4) show that brazilin has a binding affinity of −9.1 kcal/mol. In contrast, the cocrystal ligand and two other candidates, alizarin and morindone, have a binding affinity of −8.4 kcal/mol (Figure 2). The results showed that brazilin has synergistic activity with cocrystal ligands. Figure 2 presents a visualization of the best molecular docking results between PCSK9 and herb-specific active compounds. The smaller binding affinity score indicates that the bond between the ligand and the receptor is stronger, and the bond between the compound and the receptor is said to be good if the value is less than −6.0 kcal/mol [29, 34, 35].

The high binding affinity of brazilin on PCSK9 is due to the brazilin complex being mediated by a greater number of hydrogen bonds than other compounds, including cocrystal ligands. The PCSK9-brazilin complex is mediated by 7 hydrogen bonds (at amino acid residues Trp461, Arg357, Ala328, Ala330, Thr335, Asp360, and Arg412); besides hydrogen bonds there are also 2 Pi-Alkyl bonds (at residues Val460, Ala478, and Cys358), and 1 Pi-Sigma bond (on residue Pro331) which can affect bond stability. In the PCSK9-Moride complex, it is mediated by two hydrogen bonds (in amino acids Val333 and Thr 335), and 3alkyl pi bonds in amino acids Val460, Pro331, and Cys358. In Alizarin, it is mediated by three hydrogen bonds (at amino acids Pro331, Val333, and Asp360) and two pi-alkyl bonds at amino acids Val460 and Cys358. Meanwhile, in the PCSK9-ligand cocrystal complex, the bond is mediated by five hydrogen bonds (Tyr293, Arg357, Pro331, Cys358, and Arg458), two van der Waals bonds (Val333 and Ala475), one pi-alkyl bond (Val460), and one pi -sigma (Ala478). The presence of H bonds can increase the binding affinity as indicated by the increasingly negative binding affinity value, so that the more hydrogen bonds, the stronger the receptor-ligand interaction [36].

This suggests that brazilin has a stronger binding affinity with the PCSK9 receptor compared to the other potential inhibitors, including the known PCSK9 inhibitors. This stronger binding affinity can result in a more significant inhibitory effect against PCSK9, making brazilin a promising candidate for drug development. Furthermore, the interaction between brazilin and the PCSK9 receptor was found to be mediated by hydrogen bonds, which can lead to strong inhibitory interactions. This highlights the potential of brazilin as a natural-derived small molecule PCSK9 inhibitor with strong inhibitory properties against PCSK9 and its potential role to disrupt the PCSK9-LDLR interaction. To accurately predict desirable compounds, multiple selection processes must typically be applied using various docking programs such as AutoDock Vina27 or Glide28. However, as our primary objective was to obtain a candidate compound for further *in vitro* and *in vivo* experimental evaluation, we did not complete these comprehensive processes.

### 3.3. PCSK9-LDLR Binding Assay

**Figure 3:**
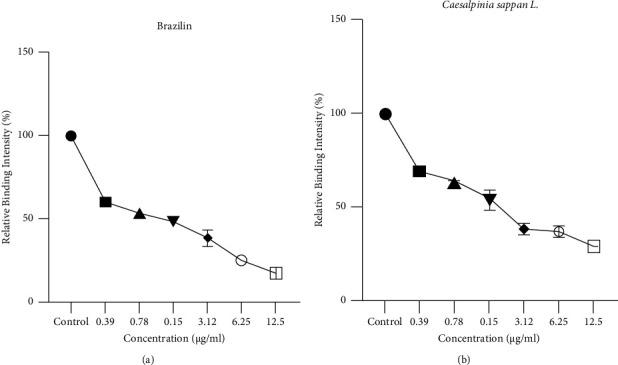
The binding of PCSK9 to the EGF-AB domain of LDLR is inhibited in a dose-dependent manner by (a) brazilin and (b) *Caesalpinia sappan* L., respectively. The inhibition of PCSK9 binding to LDLR was measured using a circulex PCSK-LDLR *in vitro* binding assay kit from MBL International, Woburn, MA, USA, as described in the materials and methods section. The relative binding intensity was set as 100% in the presence of the vehicle (at a concentration of 0). The data presented are the mean ± SD of triplicate measurements. PCSK9, proprotein convertase subtilisin/kexin type 9 and LDLR, low-density lipoprotein receptor.

Brazilin is a natural compound found in *Caesalpinia sappan* that has shown potential in binding with PCSK9, as observed through our *in silico* studies. Subsequent *in vitro* PCKS9-LDLR binding assay was conducted and the results showed that both brazilin and *Caesalpinia sappan* extract exhibited strong inhibition of PCSK9-LDLR interaction, with percentage inhibition of 82.38% and 70.64%, respectively, at a concentration of 12.50 *μ*g/mL (Figure 3). This inhibitory effect was dose-dependent, with higher concentrations of the sample leading to higher percentages of inhibition. Brazilin displayed even stronger activity than *Caesalpinia sappan* extract, with an IC_50_ value of 0.6280 *μ*g/mL, lower than the IC_50_ value of *Caesalpinia sappan* at 0.7178 *μ*g/mL (Figure 3). These findings suggest that brazilin is an effective PCSK9 inhibitor and can disrupt its interaction with LDLR. The PCSK9 inhibitory effect of *Caesalpinia sappan* further supports the efficacy of brazilin, as it is a major active compound extracted from *Caesalpinia sappan* herb [37].

Previous studies have reported that the catalytic domain of PCSK9 directly interacts with the EGF-A domain of LDLR [13]. Therefore, the kit provides a plate that is coated with the LDL receptor EGF-A domain and the His-tagged recombinant PCSK9, which allows their interaction to be visualized. The light spectrum that is read on the spectrophotometer represents the relative amount of recombinant PCSK9 that binds to LDLR's EGF-A domain on the microplate. Our results showed that both brazilin and *Caesalpinia sappan* extract treatments can reduce the binding between PCSK9-LDLR in a dose-dependent manner. The higher the dose of treatment given, the greater the decrease in PCSK9-LDLR wavelength spectrum readings. This also indicates that brazilin's mechanism of action on PCSK9-LDLR occurs at the site of action by competitive inhibition, since the brazilin treatment was added together with recombinant PCSK9 into 96-wells. As a result, the relative amount of PCSK9-LDLR measured decreased with increasing doses of brazilin.

In addition, we also measured the IC_50_ value to determine the drug potency of administering the pure compound brazilin compared to *Caesalpinia sappan* extract. Our results showed that brazilin has a lower IC_50_ of approximately 0.6280 *μ*g/mL (2.19 *μ*M), while *Caesalpinia sappan* extract has an IC_50_ of approximately 0.7178 *μ*g/mL. The observed similar inhibitory effect between *Caesalpinia sappan* extract and brazilin suggests the presence of other potential compounds within the extract that could act as PCSK9 inhibitors, potentially working synergistically with brazilin [23, 37]. *Caesalpinia sappan* extract is known to contain various phenolic components with different structural types, including brazilin, xanthone, coumarin, chalcones, flavones, and homoisoflavonoids [37]. However, multiple studies have specifically identified brazilin as the major bioactive compound in *Caesalpinia sappan* herb extract [37]. Nevertheless, our results suggest that pure brazilin exhibit a stronger inhibitory effect on PCSK9 compared to the *Caesalpinia sappan* extract.

Various strategies are being employed to develop small-molecule PCSK9 inhibitors for the treatment of hypercholesterolemia, including the use of virtual screening approaches [21, 22, 38]. For instance, through computational docking, nilotinib, an FDA-approved drug, was identified as a small molecule inhibitor of PCSK9 [38]. *In vitro* binding experiments revealed that nilotinib exhibited inhibitory activity against the PCSK9-LDLR interaction, with an IC_50_ value of 9.8 *μ*M [38]. In another study, researchers used *in silico* methods to identify imidazole-based peptidomimetics as PCSK9 inhibitors [38]. The X-ray crystal structure of PCSK9-LDLR indicated that these protein-protein interactions are mediated by a *β*-sheet motif [38]. To disrupt these interactions, Ahamad et al. designed and synthesized an N-methyl tetraimidazole derivative (MeIm), which mimics a *β*-strand motif with similar hydrogen bond donors and side chain orientations [38]. Pharmacological assessment of MeIm demonstrated its ability to suppress PCSK9-LDLR protein-protein interactions in a dose-dependent manner, with an IC_50_ value of 11.2 *μ*M [38]. The findings discussed above shed light on the wide range of approaches being investigated for the development of small-molecule PCSK9 inhibitors [38]. These approaches include both structure-based design strategies and subsequent *in vitro* studies. Notably, in comparison to compounds such as nilotinib and MeIm, brazilin exhibited a lower IC_50_ value, indicating its greater potential as a PCSK9 inhibitor in disrupting the PCSK9-LDLR interaction.

Moreover, several PCSK9 inhibitors with varying IC_50_ values in disrupting the PCSK9-LDLR interaction have been reported. For example, a natural decapeptide produced from lupin (P5) demonstrated an IC_50_ value of 1.6 *μ*M [39]. Another recent study reported NYXPCSK9i, an orally bioavailable small-molecule PCSK9 inhibitor, which exhibited submicromolar inhibitory efficacy against the PCSK9-LDLR interaction (IC_50_ = 323 nM) [40]. Despite having a higher IC_50_ value compared to those mentioned PCSK9 inhibitors, brazilin stands out as a novel compound derived from natural products with a distinct mechanism of PCSK9 inhibition. This distinguishes it from other natural-derived PCSK9 inhibitors such as berberine, quercetin, curcumin, and soy peptide, which primarily inhibit the expression of PCSK9 [23]. Although other natural-derived PCSK9 inhibitors, including lycopene, eugenol, and resveratrol, have demonstrated binding affinity with PCSK9, their specific IC_50_ values in disrupting the PCSK9-LDLR interaction have not been reported [23]. To the best of our knowledge, this study reports brazilin as the first natural product with the potential to inhibit PCSK9 by disrupting the PCSK9-LDLR interaction in a dose-dependent manner, as evidenced by its IC_50_ value.

## 4. Conclusion

Here, we report the study about the development of naturally derived small molecule PCSK9 inhibitor targeting the protein-protein interaction between PCSK9 and the LDLR by performing *in silico* virtual screening using HerbalDB database followed by simulation of molecular docking studies and *in vitro* PCSK9-LDLR binding assay. We are the first to discover brazilin as a natural compound derived from *Caesalpinia sappan* L. herb that had a high pharmacophore-fit score and a strong affinity for PCSK9's active site to LDLR. *In vitro* experiments showed that brazilin could inhibit the PCSK9-LDLR interaction in a dose-dependent manner, with an IC_50_ value of 0.6280 *μ*g/mL or 2.19 *μ*M. Due to limited research resources, a comprehensive examination of brazilin's cellular processes in inhibiting the PCSK9-LDLR interaction, including an *in vitro* kinase assay, was not carried out. Moreover, future research should aim to provide direct evidence of the ligand-binding site. One recommended approach is the use of site-directed mutagenesis on PCSK9, coupled with techniques such as co-immunoprecipitation and Western blot assays. Additionally, further evidence needs to be provided regarding brazilin's usefulness, such as whether it binds to PCSK9 directly, its specific-mediated effects, and whether it is safe for patient application. In order to assess the physiological impact of brazilin in reducing LDL-c levels, it is essential to conduct subsequent experiments, including an *in vitro* LDL uptake assay using hepatocyte cell lines, as well as *in vivo* animal studies to evaluate its efficacy. Nevertheless, our findings suggest that the approach of conducting *in silico* and *in vitro* studies could be a useful modality for discovering small molecules capable of blocking the interaction between PCSK9 and LDLR, thus potentially aiding in the development of new therapies for lipid-lowering agent. Our study is the first to report the potential of brazilin from *Caesalpinia sappan* herb as a natural compound for the development of novel PCSK9 inhibitor, which could lead to new hypercholesterolemia drugs by disrupting the interaction between PCSK9 and LDLR.

## Figures and Tables

**Table 1:** Comparison of standard setting results with best optimization results.

The best feature pharmacophore	AUC_100%_	EF_1%_	EF_5%_	Sensitivity	Specificity
Default setting result	0.83	5.,0	12.0	0.857	0.309
Best optimization results	0.93	34.0	6.0	1.00	0.79

**Table 2:** Candidate compounds of virtual screening results.

No	Candidate compounds	Molecule weight	Pharmacophore features	Pharmacophore-fitscore	Herb
1	*Morindone*	C_15_H_10_O_5_	2AR, 1H	35.34	*Morinda citrifolia* L
2	*Gentisin*	C_14_H_10_O_5_	2AR, 1H	35.33	*Gentiana lutea* L
3	*Mesuaxanthone A*	C_14_H_10_O_5_	1AR, 1H, 1HBD	35.22	*Mesua ferrea* L
4	*Beta-phenethylamine*	C_8_H_12_N	1AR, 1H, 1HBD	35.2	*Sida cordifolia* L
5	*Brazilin*	C_16_H_14_O_5_	1AR, 1H, 1HBD	35.19	*Caesalpinia sappan* L
6	*Pterofuran*	C_16_H_16_O_5_	2AR, 1H	35.11	*Pterocarpus indicus* willd
7	*N-cis-feruloyltyramine*	C_18_H_10_O_5_	1AR, 1H, 1HBD	35.07	*Hibiscus tiliaceus* L
8	*Bethanidine*	C_10_H_17_N_3_	1AR, 1H, 1HBD	35.03	*Alternanthera sessilis* (L.) R. Br. ex DC
9	6-*Methoxykaempferol*	C_16_H_14_O_7_	1AR, 1H, 1HBD	34.98	*Carthamus tinctorius* L
10	*Gartanin*	C_23_H_24_O_6_	2AR, 1H	34.98	*Garcinia mangostana* L
11	*Alizarin*	C_14_H_8_O_8_	2AR, 1H	34.95	*Rubia cordifolia* L

AR: aromatic ring, HBA: hydrogen bond acceptor, H: hydrophobic region, HBD: hydrogen bond donor.

**Table 3:** Analysis of physicochemical properties of candidate compounds as a result of virtual screening.

No	Candidate compounds	Molecule weight (g/mol)	Total HBA	Total HBD	Rotatable bonds	LogP
1	*Morindone*	270.0528	5	3	0	1.88722
2	*Gentisin*	258.0528	5	2	1	2.366
3	*Mesuaxanthone A*	258.0528	5	2	1	2.366
4	*Beta-phenethylamine*	122.0964	1	3	2	0.471
5	*Brazilin*	286.0841	5	4	0	1.6149
6	*Pterofuran*	288.0998	5	2	3	2.7912
7	*N-cis-feruloyltyramine*	313.1314	5	3	6	2.4785
8	*Bethanidine*	183.1735	3	2	2	1.3615
9	6-*Methoxykaempferol*	318.074	7	4	2	1.8693
10	*Gartanin*	408.2512	6	4	4	1.7513
11	*Alizarin*	240.0423	4	2	0	1.8732

HBA: hydrogen bond acceptor. HBD: hydrogen bond donor.

**Table 4:** Docking results candidate compound.

Candidate compound	PubChem CID	Binding affinity (Kcal/mol)
*Brazilin* ^*a*^	73384	−9.0
*Morindone* ^*a*^	442756	−8.7
*Ligand cocrystal* ^*b*^	139592542	−8.4
*Alizarin* ^*a*^	6293	−8.4
*Gartanin* ^*a*^	5281633	−8.1
*Mesuaxanthone* ^*a*^	5281651	−8.0
*Methoxykaempferol* ^*a*^	5377945	−7.7
*Gentisin* ^*a*^	5281636	−7.7
*n-Cis-feruloyltyramine* ^*a*^	6440659	−7.7
*Pterofuran* ^*a*^	44260113	−7.4
*Bethanidine* ^*a*^	2368	−6.6
*Beta phenetylamine* ^*a*^	1001	−5.1

^*a*^Candidate compound. ^*b*^Ligand cocrystal.

## Data Availability

The data used to support the findings of this study are available upon reasonable request from the corresponding author.
